# A Case of Extensive Emphysema, Produced by a Wound of the Trachea

**Published:** 1844-11

**Authors:** J. G. Girdner

**Affiliations:** Galena, Illinois


### Art. III.—*A Case of Extensive Emphysema, produced by a wound of the Trachea.* By J. G. Girdner, of Galena, Illinois.

Henry Foley, a boy aged 5 years, September 2d, was standing on a pile of barrel headings with a pocket knife in his hand, when the piece on which he stood slipped backwards and threw him on his face, and on the point of the knife—the knife penetrating the neck immediately above the left clavicle, about six lines from its junction with the sternum, and passing backwards and rather downwards wounding the trachea. The external wound was nine or ten lines in length—the blade of the knife was about two inches in length and three-fourths of an inch in width tapering to a point. I saw the boy a few minutes after the accident and found him nearly in the following situation. Emphysema over the whole body; even the head and face swelled until the eyes were closed; great difficulty of breathing, coughing violently and expectorating blood in considerable quantities; air and blood escaped by the wound at every breath, extremities cold, pulse quick and very weak. I examined the spot where he fell and found that he had bled very freely at first—but the bleeding had nearly ceased when I saw him. At the time of the accident he had hooping cough, and was, I think, in the third week of the disease. After applying a piece of adhesive plaster to the wound, and ordering stimulants to the extremities, I waited a few minutes for the violence of the cough to subside, and then proceeded to dress the wound. After sponging the blood away I closed the wound with one interrupted suture, passing the needle through the skin and cellular substance only; strips of plaster, dry lint, a compress bandage, then completed the dressing I saw him again the same day in the evening, when I found that reaction was established, the skin having become warm and moist, and the pulse full. I ordered him to be kept as quiet as possible, and enjoined a very abstemious diet.

3d. This morning I found him comfortable, with a pulse at 108; cough had been troublesome through the night, but otherwise he had rested well. The physical signs differed nothing from hooping cough; a little air still escaped from the wound during the paroxysms of coughing. The expectoration was still streaked with blood.

4th. I found him with rather more fever, for which I ordered sulphate of magnesia, which operated through the day, and gave him some relief. No air has escaped from the wound since 12 o'clock last night. Emphysema spreading to the extremities.

5th. This morning I found him much the same. No more blood expectorated; appetite good. Ordered low diet, and the patient to be kept quiet as usual.

6th. Found him still improving, the dressings still in their places, and that he was with difficulty kept in bed.

7th. To-day he has no fever, and the cough is less troublesome. Mucous rhonchus is sometimes heard in one part of the chest, and sometimes in another.

8th. Found him quite well, and the dressings having become loose I cut the stitches and removed it, when I found the wound entirely healed, except a small scab where the thread had been. I passed by the house in the evening and saw him at the cooper's shop, a distance of 30 yards from the house; when he saw me, fearing lest I should scold, he ran to the house as nimbly as usual. The emphysema had not entirely disappeared, but was disappearing slowly, and is now quite gone. The hooping cough has nearly left him; it has been much less troublesome since his wound healed, than it has been to his little brother and sister taken at the same time with him. Whether the abatement of the hooping cough was owing to the loss of blood at the time of the accident, or to the low diet and purgative afterward, or both, I will leave to older and better pathologists than myself to decide. The boy is now entirely well, and the wound in his neck was completely healed in seven days from the time of its reception.

Dr. Buck, of Louisville, who saw this case on the fifth day of the accident found that upon pressure over the scalp, the face, the chest, the abdomen, and extremities, the crepitation was as distinct as in a healthy lung; and remarked that so far as his observation extended, the case was entirely unique as to the extent of the emphysema.

Galena, Sept. 27, 1844.

